# Electrical synapses regulate both subthreshold integration and population activity of principal cells in response to transient inputs within canonical feedforward circuits

**DOI:** 10.1371/journal.pcbi.1006440

**Published:** 2019-02-25

**Authors:** Tuan Pham, Julie S. Haas

**Affiliations:** Department of Biological Sciences, Lehigh University, Bethlehem, Pennsylvania, United States of America; Brandeis University, UNITED STATES

## Abstract

As information about the world traverses the brain, the signals exchanged between neurons are passed and modulated by synapses, or specialized contacts between neurons. While neurotransmitter-based synapses tend to exert either excitatory or inhibitory pulses of influence on the postsynaptic neuron, electrical synapses, composed of plaques of gap junction channels, continuously transmit signals that can either excite or inhibit a coupled neighbor. A growing body of evidence indicates that electrical synapses, similar to their chemical counterparts, are modified in strength during physiological neuronal activity. The synchronizing role of electrical synapses in neuronal oscillations has been well established, but their impact on transient signal processing in the brain is much less understood. Here we constructed computational models based on the canonical feedforward neuronal circuit and included electrical synapses between inhibitory interneurons. We provided discrete closely-timed inputs to the circuits, and characterize the influence of electrical synapse strength on both subthreshold summation and spike trains in the output neuron. Our simulations highlight the diverse and powerful roles that electrical synapses play even in simple circuits. Because these canonical circuits are represented widely throughout the brain, we expect that these are general principles for the influence of electrical synapses on transient signal processing across the brain.

## Author summary

The roles that electrical synapses play in neural oscillations, network synchronization and rhythmicity are well established, but their roles in neuronal processing of transient inputs are much less understood. Here, we used computational models of canonical feedforward circuits and networks to investigate how electrical synapses regulate the flow of transient signals passing through those circuits. We show that because the influence of electrical synapses on coupled neighbors can be either inhibitory or excitatory, their role in network information processing is heterogeneous, and powerful. Because electrical synapses between interneurons are widespread across the brain, and in addition to a growing body of evidence for their activity-dependent plasticity, we expect the effects we describe here to play a substantial role in how the brain processes incoming sensory inputs.

## Introduction

Electrical synapses are prevalent across many brain regions, including thalamus, hypothalamus, cerebellum, and the neocortex [1–3]. In contrast to neurotransmitter-based synapses, electrical synapses are a mode of intracellular communication that transmits signals almost instantaneously, and without inactivating. Because signals cross two cell membranes, the net effect of an electrical synapse is that of a lowpass filter [3–5]: spikes are heavily attenuated, while longer or slower events, such as bursts, subthreshold rhythms, and the depolarizations that lead to spikes, are more readily shared between cells. Further, because the signal delivered is proportional to the signed difference between membrane potentials of coupled neurons, electrical synapses can exert either inhibitory or excitatory effects on a coupled neighbor, by increasing leak at rest or by transmitting activity such as post-spike hyperpolarizations, depolarizations or spikelets in either direction. A growing body of work has demonstrated ways in which electrical synapses can be modulated or modified by either synaptic [6–11] or spiking [12, 13] forms of neuronal activity.

The roles of electrical synapses in neuronal signal processing have mainly been explored in terms of their contributions to or regulation of synchrony of ongoing oscillations [14–20]. Studies focusing on the influence of electrical synapses on transient signals as they traverse the brain are fewer, but hint at specific and potentially powerful roles. For instance, propagation of spike afterhyperpolarizations through electrical synapses acts to reset and desynchronize regular firing in coupled cerebellar Golgi neurons [21]. Electrical synapses accelerate timing of spikes elicited near threshold in coupled thalamic reticular neighbors by tens of milliseconds [22, 23]. In coupled cerebellar basket cells, electrical synapses enhance and accelerate recruitment for coincident or sequential inputs [24]. Axonal gap junctions between neurons in the fly visual stream aid efficient encoding of the axis of rotation [25]. Our previous work focused on the impact of electrical synapses on transient signals in the thalamacortical relay circuit, showing that electrical coupling between inhibitory neurons leads to increased separation of disparately-timed inputs while facilitating fusion of closely-timed inputs [26].

**Fig 1:**
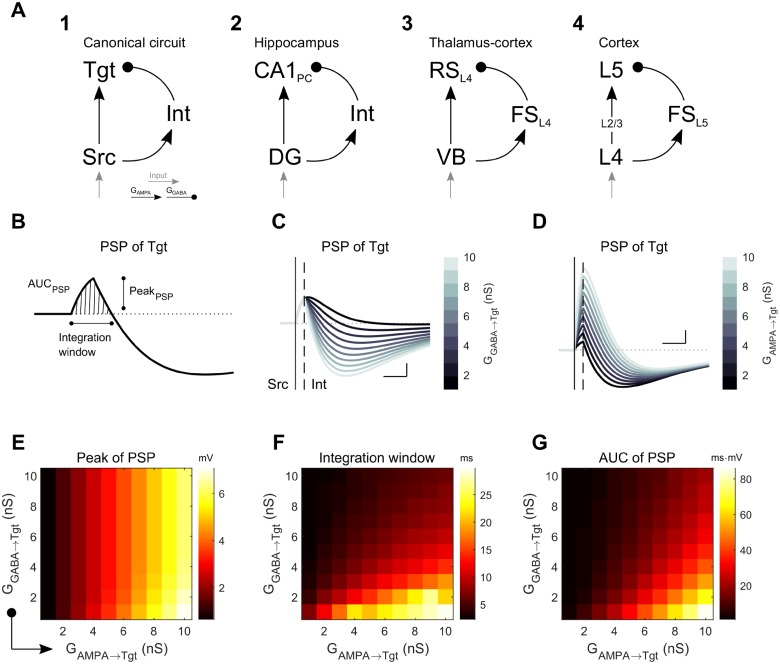
Simple canonical model (SCC) of feedforward inhibition. **A**: The Three-cell circuit model used herein (A1) with feedforward disynaptic inhibition between excitatory source (Src) and target (Tgt) neurons. This canonical model represents those found in, for example (A2) the hippocampal circuit, between dentate gyrus (DG) and CA1 cells [28]; (A3) from thalamic VB relay neurons to regular spiking cells in the somatosensory thalamocortical circuit [29]; and (A4) the cortical translaminar inhibitory circuit [30]. **B**: Example compound subthreshold postsynaptic membrane potential (PSP) in the Tgt neuron following a spike in Src, and the quantifications (PSP peak, integration window, and area under the PSP curve (AUC)) used throughout the text. **C**: Effect of different inhibitory strengths G_GABA→Tgt_ on the compound PSP in Tgt; G_AMPA→Tgt_ was 3 nS. **D**: Effect of varied G_AMPA→Tgt_ on the compound PSP of Tgt; G_GABA→Tgt_ was 6 nS. For both C and D, scale bar is 1 mV, 5 ms; the vertical straight line and dashed line mark the spike times of Src and Int, respectively. **E-G**: Combined effects of both excitatory and inhibitory synaptic strengths towards the peak, duration of the integration window and AUC of the positive portion of the compound PSP in Tgt.

In order to generalize a role for electrical synapses and variations in their strength in neuronal information processing, here we considered the canonical microcircuit, wherein two principal neurons, connected by an excitatory synapse, are also connected by disynaptic feedforward inhibition (Fig 1A_1_)[27]. This circuit motif reappears through the brain in areas ranging from the hippocampal CA1 pyramidal neurons [28], somatosensory L4 cortical neurons receiving inputs from the ventrobasal complex [29], and the cortical translaminar inhibitory circuits [30] (Fig 1A_2-4_). Starting with a canonical circuit, we progressively expanded models and analysis from a single circuit to a network composed of canonical circuits. We provided these models with closely timed inputs, in order to determine how the embedded electrical and inhibitory synaptic connections between interneurons influence subthreshold integration and spiking statistics at the output stage of the model. Our simulations demonstrate that electrical synapses enable a high degree of specificity and diversity of processing of transient signals for both subthreshold activity and network activity. Because electrical synapses are widespread throughout the mammalian brain, we expect that these are principles that apply widely to neuronal processing of newly incoming information as it passes through the brain.

## Results

### Subthreshold integration in canonical circuits

We started our inquiry by creating a three-cell circuit composed of Izhikevich-type neurons (see Methods) to represent the canonical neuronal microcircuit: two excitatory neurons, with an interneuron providing feedforward inhibition (the simple canonical circuit (SCC), Fig 1A). Upon excitation of the source (Src) neuron, this model produces a compound postsynaptic potential (PSP) in the target (Tgt) neuron that is a sum of a purely excitatory PSP from the Src neuron and an inhibitory PSP arriving with a delay from the inhibitory interneuron (Int). The features of the compound PSP (Fig 1B)–its peak amplitude, its net total excitation (area under the positive component of the PSP curve, or AUC), and the duration of the integration window–together determine whether Tgt will generate a spike given sufficient input. The PSP depends predictably on the strength of the PSPs arriving from Int (Fig 1C) and Src (Fig 1D); generally, inhibition curtails the excitation, while the Tgt PSP peak is proportional to excitation from Src (Fig 1E). More specifically, in feedforward circuits G_GABA→Tgt_ does not limit the PSP peak (Fig 1E), but increases in G_GABA→Tgt_ do limit the integration window (Fig 1F) and the net total excitation (Fig 1G). Thus, the interneuron limits the overall excitation and possibility of Src triggering an action potential in Tgt.

**Fig 2:**
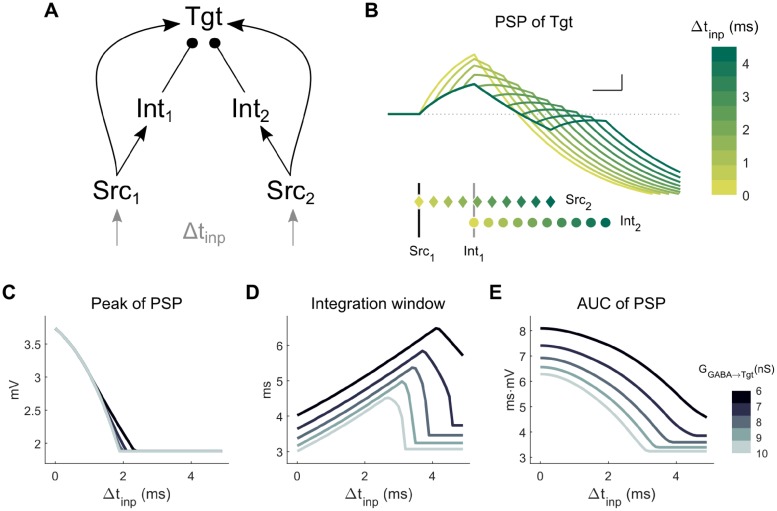
Coupled canonical circuit (CCC) model: Two Src neurons and two Int neurons lead to a common Tgt neuron. **A**: Model schematic. For the simulations shown here, there were no connections between the Int neurons. Each Src neuron received its own input, with timing difference between the two inputs Δt_inp_. **B**: PSPs in Tgt for varied Δt_inp_, with a color code representing different values of Δt_inp_. Spike times are shown below for Src_1_ (vertical black line), Src_2_ (colored diamonds ♦), Int_1_ (gray line) and Int_2_ (colored circles ●), each vertically separated for clarity. G_GABA→Tgt_ was 8 nS. Scale bar is 1 mV, 1 ms. **C-E**: Integration parameters for the Tgt PSP as defined in Fig 1 for varied combinations of Δt_inp_ and G_GABA→Tgt_.

To understand how Tgt might sum input from multiple sources, our next step towards building larger models was to couple two canonical circuits, using two Src neurons and two Int neurons leading to a common Tgt (the coupled canonical circuit (CCC), Fig 2A). Using the CCC, below we explore the effects of varied connection types between the Int neurons.

To start, in the absence of connectivity between Int neurons (Fig 2A), we provided both Src neurons with brief inputs sufficient to evoke single spikes in the Src neurons while varying the time delay between the inputs Δt_inp_. From these simulations, we observed that the inhibition from the Int neurons limited summation of the two Src signals in Tgt (Fig 2B). First, we noted that as for the SCC (Fig 1), the peak of the Tgt PSP is preserved across large ranges of G_GABA→Tgt_ (Fig 2C), as it mainly depends on the delay Δt_inp_. The integration window and AUC depend on both the delay Δt_inp_ and the strength of inhibition. Increases in G_GABA→Tgt_ curtailed the integration window and AUC of integration in the Tgt PSP (Fig 2D and 2E), diminishing these measures in a monotonic and straightforward manner. These results provide a baseline of expectations for the following simulations in which the Int neurons are connected by electrical and inhibitory synapses.

**Fig 3:**
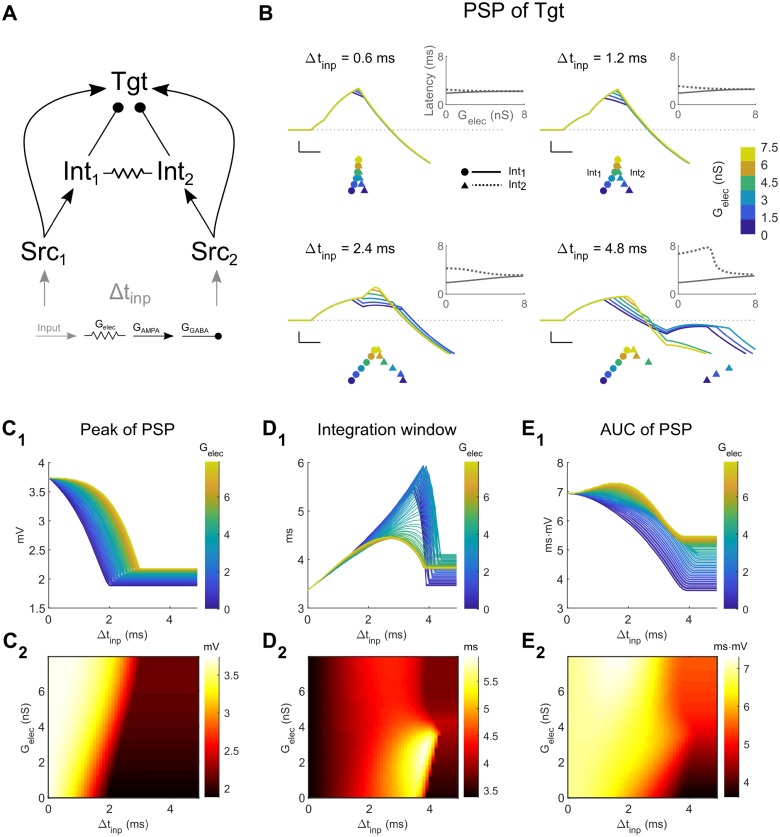
Coupled canonical circuit (CCC) model: Two Src neurons and two Int neurons lead to a common Tgt neuron. **A**: Model schematic. For the simulations shown here, the Int neurons were electrically coupled. Each Src neuron receives its own input, with timing difference between the two inputs as Δt_inp_. **B**: Examples of Tgt PSPs for different electrical synapse strengths between interneurons in the network (colored lines and legends). Each subpanel represents different values of input timing differences Δt_inp_. Scale bar is 1 mV, 1 ms. Colored symbols below PSPs represent the spike times of Int_1_ (circle ●) and Int_2_ (triangle ▲). Symbols are vertically separated for clarity. Insets show latencies of Int_1_ (solid lines) and Int_2_ (dashed lines) spikes relative to Src_1_, against G_elec_. **C-E**: Increased electrical coupling leads to increased peak and AUC of the Tgt PSP, and decreased the integration window.

Next, we included an electrical synapse between the two Int neurons of the CCC (Fig 3A). We limited the range of strength of the electrical synapse to vary between 0 (uncoupled) and a coupling coefficient of ~0.3, which represents common strengths found in the thalamus [13, 31] and cortex [32–34]. We again provided this circuit with identical inputs, with varying time delay between the inputs Δt_inp_ (Fig 3B). As electrical synapse strength increased we noted increased delays in Int_1_ spiking due to increased leak from the electrical synapse, and we also noted accelerations in Int_2_ spiking due to the excitatory spikelet it received from Int_1_ (Fig 3B, rasters and insets). Together, these changes in Int spike times result in a net synchronizing effect on summed Int inhibition for electrical coupling in this regime of input timing. As a result, within the CCC, electrical coupling enhanced Tgt input integration for closely timed inputs by allowing for increased PSP peaks (Fig 3C) and AUC (Fig 3E), while narrowing the integration window (Fig 3D) for the PSP. In the same circuit, for more than ~4 ms of Δt_inp_, small values of electrical synapse strength only served to increase leak in Int_2_ well after the spikelet had finished, ultimately delaying its spike (Fig 3B, lower right). Increases in electrical synapse strength, however, allowed for the spikelet from Int_1_ to directly elicit spiking in Int_2_, which spiked earlier than it might have otherwise (Fig 3B, lower right). The net effect in this range of larger input timing allows the PSP in Tgt to increase by small amounts in peak amplitude (Fig 3C, Δt_inp_>4ms), but the shortened integration window (Fig 3D, Δt_inp_>4ms), resulting from the earlier spike in Int_2_, effectively prevents summation of the two Src inputs in Tgt. Thus, the varied effects of increased leak or excitatory spikelets between Int neurons resulting from an electrical synapse with varied strengths increases flexibility for responses to signals passing through this version of the CCC, as compared to the CCC with no connections between the Int neurons (Fig 2).

**Fig 4:**
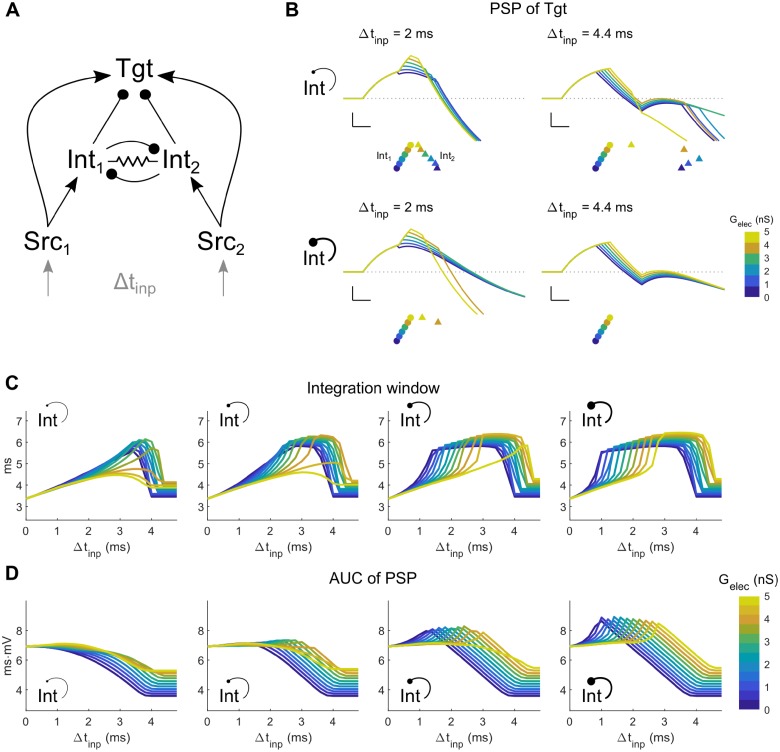
Coupled canonical circuit (CCC) model: Two Src neurons and two Int neurons lead to a common Tgt neuron. **A**: Model schematic. For the simulations shown here, the Int neurons were electrically coupled and were reciprocally connected by an inhibitory synapse. Each Src neuron receives its own input, with timing difference between the two inputs Δt_inp_. **B**: Examples of Tgt PSP for different electrical synapse strengths between interneurons of the coupled network (colored lines and legends). Each subpanel shows PSPs for different input timing differences Δt_inp_ (left: 2 ms, right: 4.4 ms), and strength of reciprocal inhibition G_GABA→Int_ (top: 1 nS, bottom: 7 nS). Scale bar is 1 mV, 1 ms. Colored symbols represent the spike times of Int_1_ (circles ●) and Int_2_ (triangle ▲), with colors representing different values of G_elec_ between the two interneurons. Symbols are vertically separated for clarity. **C-D**: Integration window and AUC of the PSP in Tgt for varied strengths of G_elec_ (0–5 nS) and G_GABA→Int_ (1, 3, 5, 7 nS from left to right).

While GABAergic coupling is rare between nearby electrically coupled inhibitory neurons of the thalamus [31, 35], it is sometimes observed between coupled pairs of inhibitory interneurons in cortex [32–34, 36]. To test the additional effects of GABAergic connectivity between electrically coupled interneurons, we included symmetrical GABAergic synapses between Int neurons in the CCC model (Fig 4A). From these simulations, we see that for transient inputs separated by Δt_inp_, the additional synapse further expanded the possibilities for subthreshold summation of inputs in Tgt. While the effect of G_GABA→Int_ on the peak PSP in Tgt (Fig 4B) was not substantially different from the CCC without an inhibitory synapse, the integration window expanded with stronger inhibition (Fig 4C), and the AUC of the PSP also increased for stronger G_GABA→Int_ (Fig 4D). Further, in the presence of stronger reciprocal inhibition, increased electrical coupling shifted the maxima in Tgt integration windows and AUCs rightwards, towards larger values of Δt_inp_ (Fig 4C and 4D, right columns). Thus, comparing Figs 3C–3E, 4C and 4D, the interaction between electrical and inhibitory synapses is nonmonotonic and complex. In particular, we note that strong inhibition competes with electrical synapses alone in terms of the impact on PSP integration window for closely timed inputs: increases in electrical synapse strength shorten the window in the context of weak inhibition, while stronger inhibition broadens the window, especially for weaker electrical synapses (Fig 4C, left to right). However, for larger Δt_inp_, both types of interneuron coupling broadened the window.

Thus, similar to our previous demonstration [26], we note that electrical synapses act directly on inhibitory interneurons and indirectly through inhibitory synapses onto a target in diverse ways to control the processing of transient signals passing through a neuronal circuit. We also note that changes in electrical synapse strength can potentially halve or double the PSP (Fig 3C_1_), the integration window (Figs 3D_1_ and 4C), or area under the curve (Figs 3E_1_ and 4D). Thus, modulation [8–10] or activity-dependent modifications of electrical synapses [12, 13] potentially exert powerful impacts on subthreshold summation of transient inputs in the Tgt cell, and on canonical neuronal circuits.

### Spiking responses in networks of canonical circuits

**Fig 5:**
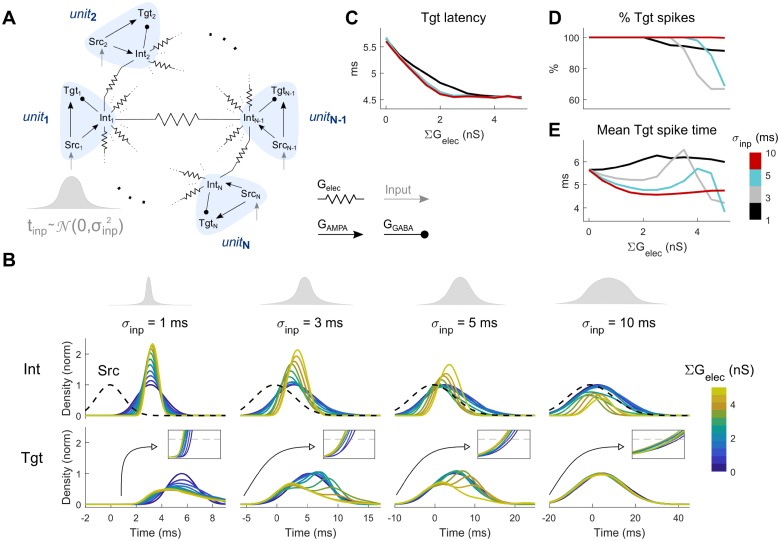
Coupled canonical network (CCN), comprising subunits of SCCs. **A**: Model schematic. For the simulations shown here, the Int neurons were connected by electrical synapses. Each Src neuron receives a single input, with arrival times drawn from a Gaussian distribution with specific standard deviation σ_inp_. **B**: Normalized distributions of the input (top) and distributions of spike times in the Int (middle) and Tgt (bottom row) populations. Each column represents a different value of input timing distribution standard deviation (σ_inp_ = 1, 3, 5, 10 ms, from left to right). Dashed line (middle row) represents the average distribution of Src population spike times centered around t = 0 ms. Insets (bottom row) highlight the latencies of Tgt population, with threshold used to determine them (grey dashed line, 0.1). Line colors represent different values of electrical coupling strength of the interneuron population. **C**: Latency of Tgt distributions relative to Src, computed by thresholding the distributions in at 0.1. **B**. **D**: Response rate of Tgt neurons shown for each σ_inp_, with 100% indicating spikes in all 50 Tgt neurons. **E**: Average Tgt spiking time (center of Tgt distribution) relative to Src, shown for each σ_inp_.

To study responses of a population of Tgt neurons, we embedded 50 units of the canonical circuit into a network (the coupled canonical network (CCN), Fig 5A), and started our analysis with electrical coupling between the Int neurons. In order to study spiking rather than subthreshold activity in the Tgt population, we increased G_AMPA_ from the Src to the Tgt (G_AMPA→Tgt_) and decreased G_AMPA_ from Src to Int (G_AMPA→Int_) in each unit, along with modest increases to Tgt excitability (see Methods) in order to elicit spiking in the Tgt neurons within 5–6 ms of Src spiking, latencies that are consistent with latency to input in the regular spiking neurons in hippocampus [37]. To each Src neuron in the layer of 50, we provided identically sized inputs drawn from Gaussian distributions of input times with a standard deviation of σ_inp_ (Fig 5B). We then quantified the distribution of spike times in the Int and Tgt populations (Fig 5B). From these results, we observed that increases in electrical synapse strength acted to narrow and delay the distributions of spike times in the Int layer (Fig 5B, middle row), and markedly increased maximal spiking density for smaller σ_inp_. In the Tgt population, the narrowed Int distributions that resulted from increased electrical coupling allowed some Tgt neurons to spike earlier, hence decreasing the latency of Tgt population from the input (Fig 5B, bottom row and insets; Fig 5C). Increased electrical synapse strength also decreased total spiking in Tgt (Fig 5D), in fact selectively reducing later spikes and thereby shifting mean Tgt spike times towards smaller latencies (Fig 5E), as a result of changes in Int spiking.

**Fig 6:**
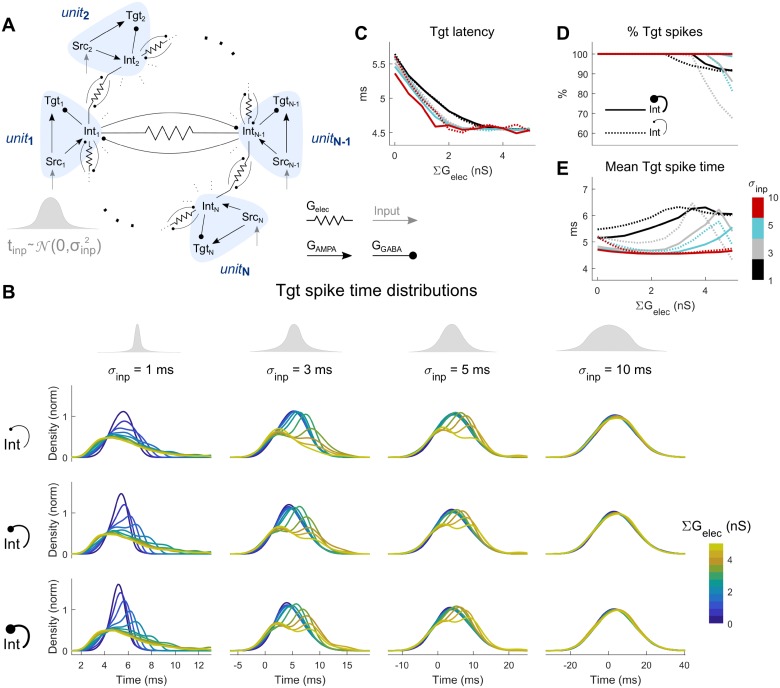
Coupled canonical network (CCN), comprising subunits of SCCs. **A**: Model schematic. For the simulations shown here, the Int neurons were connected by electrical synapses and reciprocal inhibition. Each Src neuron receives its own input, with arrival times drawn from a Gaussian distribution with specific standard deviation σ_inp_. **B**: Normalized spike time distributions of the Tgt population. Each subpanel represents a different combination of input timing distribution standard deviation (σ_inp_ = 1, 3, 5, 10 ms, from left to right) and reciprocal inhibition strength (∑G_GABA→Int_ = 1, 3, 5 nS from top to bottom). **C**: Latency of Tgt distributions relative to Src, computed by thresholding the distributions in **B** at 0.1, shown here for weak (∑G_GABA→Int_ = 1, dotted lines) and strong (∑G_GABA→Int_ = 5, solid lines). **D**: Response rate of Tgt neurons shown for each σ_inp_ and for weak and strong inhibition, as in **C**, with 100% indicating spikes in all 50 Tgt neurons. **E**: Average Tgt spiking time (center of Tgt distribution) relative to Src, shown for each σ_inp_ and for weak and strong inhibition, as in **C**.

Finally, in addition to electrical coupling, we included GABAergic connectivity between neighboring Int pairs of the CCN (Fig 6A and 6B). The effects of electrical synapses on this network were similar to the previous model (Fig 5): increases in electrical synapse strength decreased latency (Fig 6C), decreased total spiking in Tgt (Fig 6D), and selectively reduced later spikes, but here shifting its distributions towards later times overall (Fig 6E). Increased reciprocal inhibition was most effective for small values of σ_inp_ (Fig 6B, left column), where stronger inhibition between Int neurons allowed Tgt neurons to spike more often (Fig 6D, solid lines) and somewhat earlier (Fig 6E, solid lines), thus effectively counteracting the effect of electrical synapses.

**Fig 7:**
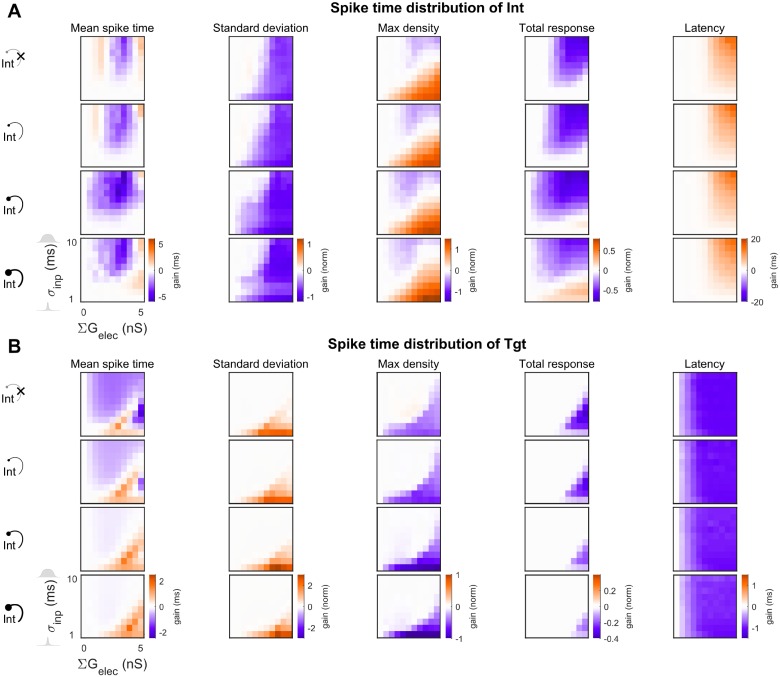
Changes in properties of spike train distribution’s of Int and Tgt for the CCNs. Values are expressed as normalized to the input (Src) distributions. (**A**) Int population and (**B**) Tgt population. In each panel, rows represent increasing reciprocal inhibitory strength within the Int population (ΣG_GABA→Int_ = 0, 1, 3, 5 nS, from top to bottom). The top row of each set is the baseline CCN with ΣG_GABA→Int_ = 0 (Fig 5), while the second, third and fourth rows represent the CCN with ΣG_GABA→Int_ > 0 (Fig 6). The first column of each heat map always represents the uncoupled case, with 0 gain as indicated in white (see Methods). Within each heat map, electrical coupling ΣG_elec_ is varied on the x axis and input distribution size σ_inp_ is varied on the y axis. Latency is shown relative to Src.

We compared the behavior of the CCN with and without reciprocal inhibition by plotting the change in spiking properties due to electrical synapses relative to the uncoupled case (∑G_elec_ = 0) across input time distributions for the Int (Fig 7A) and Tgt (Fig 7B) populations. While the input was Gaussian, the Tgt distributions were often not Gaussian; therefore, we measured mean spike times, standard deviations of spike times, maximal density and total density of spike time distributions, along with the relative latency (see Methods). We observed that most of the effects that electrical synapses exerted on the output Tgt distribution were strongest for small σ_inp_, except for latency. Mean spike times both increased and decreased for different combinations of σ_inp_ with inhibitory and electrical synapse strengths, while the spread (standard deviation) of spike times consistently increased with electrical synapse strength. Maximum density (corresponding to peak spiking) and total density (total spike count) of spiking, as well as relative latencies, decreased with increase in electrical synapse strength. Further, inclusion of larger reciprocal inhibition between the Int neurons led to decreased spiking within the Int population, thereby allowing later-activated Tgt neurons to spike faster, especially for the electrically uncoupled cases (Fig 6B, blue lines). Increased electrical coupling combined with reciprocal inhibition led to increased inhibition within the Int layer, leading to better -synchronized Int activity but decreased total responses of the Int population. As seen previously [26], the effects of electrical and inhibitory synapses within the Int layer interacted in complex ways; for one, Tgt spiking decreased less for stronger inhibition.

Together, these results show that electrical synapses embedded within a network composed of canonical circuits have powerful and heterogeneous effects on the spiking of the Tgt output population, by altering spike times and total responses properties, as inputs from Src propagate through the network.

**Fig 8:**
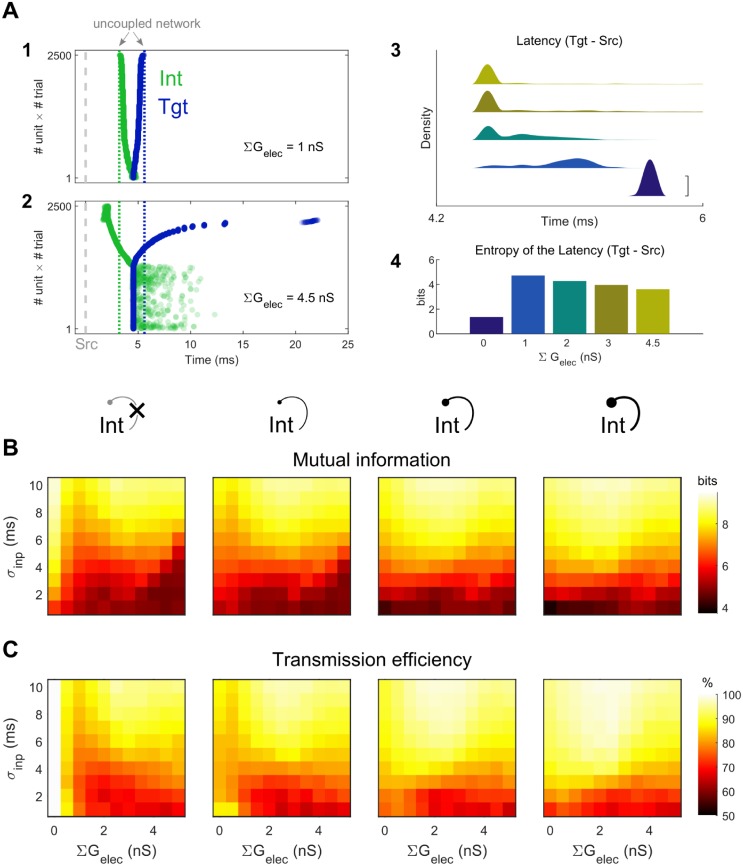
Information transfer between Tgt and Src in the CCN. **A1, 2**: Example spike rasters for one set of CCN simulations demonstrating dispersion of Int and Tgt spike times, accumulated from all 50 SCC subunits over 50 trials with σ_inp_ = 5 ms and ΣG_elec_ = 1 nS (top) or ΣG_elec_ = 4.5 nS (bottom), and without reciprocal inhibition. Gray dashed line represents Src spike times, and colored dotted lines represent the uncoupled network (no electrical nor reciprocal inhibitory coupling). Spike times are ordered by Tgt- Src latency for clarity. **A3, 4**: Smoothed distributions of Tgt latencies relative to Src (A_3_, vertically offset for clarity, scale bar = 0.05) and entropy of the latencies (A_4_; ΣG_elec_ = 0, 1, 2, 3, and 4.5 nS). **B**: Mutual information between Src and Tgt distributions, plotted against ΣG_elec_ and σ_inp_, for ΣG_GABA→Int_ = 0, 1, 3, 5 nS from left to right. **C**: Transmission efficiency (percent of Tgt entropy attributed to Src) from Src to Tgt.

We quantified the mutual information between the spike time distributions of Src and Tgt, as well as the transmission efficiency from Src to Tgt (Fig 8). For the electrically uncoupled case with no reciprocal inhibition, each Src elicited a single spike within its Tgt unit with predictable latency, leading to Tgt spike time distributions that mirrored Src distributions and resulted in maximal mutual information and 100% transmission efficiency. Increases in electrical synapse strength acted to disperse Tgt spike times (Fig 8A_1-3_) and increased the joint distribution entropy (Fig 8A_4_), and thus tended to diminish the information shared between Src and Tgt (Fig 8B). Both mutual information and transmission efficiency were modulated by ∑G_elec_ for any given input distribution, but without inhibition, neither measure recovered its peak value of ∑G_elec_ = 0 (Fig 8B and 8C, left). Transmission efficiency decreased with larger values of electrical coupling, with more notable decreases with smaller σ_inp_ (Fig 8C). The largest decrease was roughly 35%.

As interneuron reciprocal inhibition was added and spiking in the Int population decreased, some neurons within the Tgt distribution spiked much faster but with less uncertainty, creating narrower distributions and smaller entropy. As a result, both mutual information and transmission efficiency overall increased relative to the network with no inhibitory synapses (Fig 8B and 8C). For all networks with nonzero inhibitory synapses, the maximal values of mutual information and transmission efficiency occurred for ∑G_elec_ > 0.

## Discussion

Overall, our simulations together demonstrate that electrical synapses between interneurons in canonical networks regulate both subthreshold activity and network spiking activity, ultimately exerting powerful and complex effects on the output activity of the network as it processes and passes on its inputs. The general effect was that for closely-timed inputs, increases in electrical coupling strength often led to delay of spiking in the inhibitory interneurons, which enabled larger summations of source inputs in the target output and at earlier times. Yet simultaneously, increased coupling strength produced stronger synchronized inhibition from the interneuron population to the target population, at later times, which limited the output of the Tgt neurons.

These complex interactions effects highlight the diverse roles that electrical synapses of dynamically varying strength might play in the circuits that contain them across the brain. In TRN, as we have previously shown [26], electrical synapses ultimately act to either further fuse or to aid in discrimination of sensory inputs as they are passed to cortex by relay cells. Within for instance somatosensory cortex, the impacts of electrical synapses within the networks that embed canonical circuits may be similar–to sharpen the timing spread or modify gain in principal cell firing within a barrel, in response to whisker stimulation [38]. In hippocampus, electrical synapses may aid in place cell spatial precision [39].

Electrical synapses in a local network regulate subthreshold summation of inputs to a target neuron. Our simulations show that stronger electrical coupling allow the target neuron to integrate its source inputs with higher summed PSP peaks, yet limit time windows for further inputs to summate, thereby acting as a coincidence detector [40]. Furthermore, changes in electrical coupling in a local network of interneurons, as might result from activity-dependent electrical synapse plasticity, lead to more flexibility in regulating subthreshold summation. However, our results also show that reciprocal inhibition between the electrically coupled interneuron pair expanded the integration window and the area under the excited portion of the target PSP, especially for relatively large differences in input timings. Increases in reciprocal inhibition allow for widening integration windows of disparately-timed inputs, in that case acting more like an integrator. This suggests that the interactions between the electrical coupling and reciprocal inhibition within the local interneuron networks could regulate the ability for the target neuron to either be a coincidence detector or an integrator [40] for its inputs.

At a network level, we find that electrical coupling of the interneuron population modulates the target population activity over different distributions of input timings. Similar to the subthreshold effect, increase in electrical synapse strength led to a more delayed, yet denser activity in the interneuron population, effectively synchronizing its activity. Hence, stronger electrical coupling allowed stronger and earlier responses of the target layer activity, but weaker later responses. However, because the activity of the interneurons was limited within a smaller temporal window due to their electrical coupling, inhibition towards the target population was limited in time. Hence the output activity was more sustained compared to uncoupled cases. As a result, electrical coupling allowed earlier yet sparser Tgt responses, and effectively reduced both the mutual information and the transmission efficiency between Src and Tgt. This effect was strongest for small input distribution sizes. One result of this interaction is that although the integrity of Src-Tgt coding was corrupted, electrical coupling between the interneurons increased temporal heterogeneity (increased sparsity) amongst the Tgt population as inputs coming from different Src neurons arrived. In contrast, reciprocal inhibition decreased interneuron activity and thereby enhanced the target response in presence of electrical coupling. However, for closely-timed input distributions in the presence of reciprocal inhibition, the target temporal code distribution narrowed in timing spread, especially for electrically uncoupled or weakly coupled cases, resulting in loss of mutual information and transmission efficiency.

These results point toward yet another type of interaction between electrical coupling and reciprocal inhibition within the interneuron population that regulates the temporal code of the output distribution. Although both types of synapse disrupt the input-output temporal integrity of closely-timed input distributions, electrical synapses act to increase temporal heterogeneity in the output layer, while the inhibitory synapses decrease output temporal heterogeneity.

As the issue of transient neuronal signal processing in models that include electrical coupling between inhibitory neurons has been understudied, even broader implications from this work remain to be determined. Interactions between electrical synapse-transmitted excitatory spikelets and inhibitory afterhyperpolarizations showed a temporarily reduced probability of spike generation [21] that foreshadowed the results here. Recent work shows that gap junctions couple PV interneurons across barrel boundaries [41], suggesting that electrical coupling may connect broader and more complex circuits than the simple canonical circuits used here; in barrel cortex, both within and across different barrel columns. The result we have described above demonstrate urgent necessity for considering electrical synapses in simpler and more complex models of neuronal networks.

## Methods

### Model and simulation

The canonical disynaptic feedforward-inhibition network can be simplified as a small 3-cell simple canonical circuit (SCC) (Fig 1A_1_), comprising of a Src (source), an Int (interneuron) and a Tgt (target) neuron. For subthreshold investigations, we explored a small network (the CCC) composed of two canonical circuits. For activity explorations, we used a network model (the CCN) comprising subunits of canonical circuits. In this paper, we present results from N = 50 subunits and target neurons.

### Izhikevich type neuron model

For generalizability, we modelled Src and Tgt as regular spiking (RS) neurons and Int as a fast spiking (FS) neuron with Izhikevich formulism [42]. Briefly, Eqs 1 and 2 describe the dynamics of the membrane potential *v* and recovery current *u* respectively, with the spiking condition in Eq 3. Additionally, implementation of FS neuron model also differs from RS as described in Eqs 4 and 5 [42].

$$C\frac{dv}{dt}=k(v-v_{r})(v-v_{t})-u+I_{app}+I_{syn}$$ (1)

$$\frac{du}{dt}=a\times f(v,u)$$ (2)

ifv≥vp,then{v←cu←u+d (3)

$$f\left(v,u\right)=\{\begin{array}{r} b\left(v-v_{r}\right)-u\;\;\;\;if\;Type=\text{RS}\\ U\left(v\right)-u\;\;\;\;if\;Type=\text{FS} \end{array}$$ (4)

$$U\left(v\right)=\{\begin{array}{rr} 0 & if\;v<v_{b}\\ b{\left(v-v_{b}\right)}^{3} & if\;v\geq v_{b} \end{array}$$ (5)

$$I_{app}(t)=I_{hold}+I_{inp}(t)$$ (6)

We applied a holding current I_hold_ (Eq 6) of 50 pA to Int to easily evoke spiking in response to input from Src. For subthreshold investigation (SCC, CCC), we modelled Src and Tgt with the same set of parameters of an RS neuron (Table 1). To inspect network activity (CCN), we tuned the parameters (halved capacitance and lowered threshold potential) and applied a 10 pA holding current for each Tgt neuron in order for its Src to easily evoke its spiking.

**Table 1:** Izhikevich model parameters. Single asterisk * is for SCC and CCC. Double-asterisk ** is for CCN.

	Src	Int	Tgt
**Type**	RS	FS	RS
***C (pF)***	100	20	100* or 50**
***v*_*r*_*(mV)***	-60	-55	-60
***v*_*t*_*(mV)***	-40	-40	-40* or -45**
***v*_*p*_*(mV)***	35	25	35
***k (nS)***	0.7	1	0.7
***a (1/ms)***	0.03	0.2	0.03
***b (nS)***	-2	0.025	-2
***c (mV)***	-50	-45	-50
***d (pA)***	100	0	100
***v*_*b*_*(mV)***		-55	

### External input to Src neurons

In all cases, only Src received external input: a brief 20–30 ms of 200–300 pA DC input, sufficient to evoke a single action potential in in Src (Eq 7). For the SCC and CCC, we varied the arrival time differences between input to Src_2_ and input to Src_1_ as Δt_inp_ from 0 to 20 ms. For the CCN, timings of Src inputs were drawn from a normal distribution with standard deviation as σ_inp_, which we varied from 1 to 10 ms.

$$I_{inp}(t)=I_{0}\;if\;t\;\in [t_{inp},t_{inp}+d_{inp}]$$ (7)

### Synaptic connections and different network configurations

For synaptic inputs, neurons excite each other via AMPA synapses, inhibit each other via GABA synapses or couple with each other via electrical synapses, as described in Eqs 8–11. Src sends AMPA excitatory input to Tgt and Int separately sufficiently to drive Int to spike and for Tgt to receive a noticeable EPSP (SCC, CCC) or to spike (CCN). Where indicated, Int also sends GABAergic inhibitory input to Tgt. Int neurons are also connected by an electrical synapse.

$$I_{syn}(t)=I_{elec}+I_{AMPA}+I_{GABA}$$ (8)

Electrical synapses were implemented as symmetric linear resistance, as shown in Eq 9. For two coupled Int neurons, we varied the electrical synapse conductance of from 0–8 nS (unless otherwise noted), corresponding to coupling coefficients (cc) of roughly 0–0.33. For the CCN, Int neurons are electrically coupled homogeneously in an all-to-all manner (Figs 5A and 6A), with each coupling conductance scaled to the number of Int neurons as G_elec_ = ∑G_elec_ /N_Int_.

$$I_{elec}=G_{elec}\times (v_{pre}-v_{post})$$ (9)

Chemical synapses were implemented with a single exponential decay as described in Eqs 10 and 11, and implemented following the example of [43] in *Brian2* documentation. The synaptic reversal potentials and time constants were fixed: E_AMPA_ = 0 mV, τ_AMPA_ = 2 ms and E_GABA_ = -80 mV, τ_GABA_ = 10 ms. The conductance parameters were either fixed or varied as in Table 2. For the CCN where inhibition is included, the Int population also reciprocally inhibits itself in an all-to-all manner. Each inhibitory conductance was also scaled to the number of Int’s as G_GABA→Int_ = ∑G_GABA→Int_ /N_Int_.

$$I_{chem}=G_{chem}\times s_{chem}\times (E_{chem}-v_{post})\;with\;chem=\{AMPA;GABA\}$$ (10)

$$\frac{d}{dt}s_{chem}=-\frac{s_{chem}}{\tau_{chem}}+\sum_{k}\delta (t-t_{k})\;with\;t_{k}\;is\;time\;of\;presynaptic\;input$$ (11)

**Table 2:** Synaptic conductances for different network configurations.

		SCC	CCC	CCN
**Src → Int**	G_AMPA→Int_	10	10	5
**Src → Tgt**	G_AMPA→Tgt_	1–10	3	20
**Int → Tgt**	G_GABA→Tgt_	1–10	6–10	10
**Int → Int**	G_elec_ or ∑G_elec_		0–8	0–5
**Int → Int**	G_GABA→Int_ or ∑G_GABA→Int_		0–7	0–5

### Simulation environment

Simulations were run in the Python-based open source simulator *Brian2* [44]. Subthreshold simulations were run for 100 ms with dt = 0.01 ms (SCC, CCC). For each parameter set in network activity investigations (CCN), 50 random simulations were run as with external input timings to Src population drawn from a normal distribution with size σ_inp_. Each simulation was 200 ms and dt = 0.05 ms because less accuracy was required and for speed.

### Analysis

Analysis and visualization were mainly performed in MATLAB (MathWorks R2018a) and the open source graphics editor Inkscape 0.92.3.

### Subthreshold investigation

For subthreshold investigations (Figs 1–4), we obtained the net postsynaptic potential (PSP) of the Tgt neuron and quantified the peak potential, duration (or integration window) and area under the curve (AUC) of the positive portion of the PSP (Fig 1B).

### Network activity investigation

For each set of parameter θ, we obtained the raw distribution of spike times X(θ, *C*) = {X_*k*_(θ, *c*_*i*_)} population *C* aggregated from all X_*k*_(θ, *c*_*i*_), which is the spike time array of neuron *c*_*i*_ in simulation *k*^*th*^. The symbol *C* (or *c*) represents the population name, can either be any of the following {Src, Int, Tgt}. *i* = {1, 2 … *N*_*C*_} with *N*_*C*_ as the number of neurons in population *C*. *k* = {1, 2 … *N*_*s*_} with *N*_*s*_ as the number of random simulations. In this paper, we used *N*_*C*_ = 50 with all *C* and *N*_*s*_ = 50 as described earlier.

### Normalized properties of spike time distributions

To easily compare between different initial input distributions, we generally normalized all quantifications to the Src population (Figs 5–7). More specifically, for each X_*C*_ = X(θ, *C*), we defined normalized mean spike time as the difference between the mean of X_*C*_ and that of X_*Src*_. The normalized standard deviation was the standard deviation of X_*C*_ normalized over the standard deviation of X_*Src*_.

For each X_*C*_ = X(θ, *C*), we calculated the spike density from the smoothed histograms of spikes times. More specifically, each array of spike times X_*C*_ was histogrammed with a bin width that equals to one-tenth of the σ_inp_ in order to avoid under-sampling with small σ_inp_ and over-sampling with large σ_inp_; then it was smoothed by convoluting with a Hanning window of size 20 to obtain the un-normalized density *d*_*C*_(t). For visualization, the spike times were translated relative to the mean Src spike time distributions, whereas the densities were scaled over the maximum density of the Src distribution to calculate the normalized density *D*_*C*_(t). Note: neither *D*_*C*_(t) nor *d*_*C*_(t) represented estimated probability density function, because the smoothed histograms were not normalized by their number of samples.

For quantification comparison, we defined normalized maximum density as the maximum density of *d*_*C*_(t) normalized over that of *d*_*Src*_(t). The normalized total response was calculated by normalizing the area under the curve of *d*_*C*_(t) over that of *d*_*Src*_(t) (note: neither *D*_*C*_(t) nor *d*_*C*_(t) represented estimated probability density function, hence AUC was not necessarily 1). Lastly, relative latency was defined as the time point which *d*_*C*_(t) reached 10% of maximum density, relative to the same measure calculated for Src spike time distribution X_Src_.

Additionally, gain of a particular property *Q* of a spike time distribution due to a parameter set θ was defined as the difference between itself and the same property when the electrical coupling parameter in set θ equals to 0, in other words *Gain*[*Q*(θ)] = *Q*(θ)–*Q*(θ_electrically uncoupled_).

### Mutual information and transmission efficiency

For network investigation, we also quantified the mutual information and transmission efficiency between the Src and Tgt population spike time distribution (Fig 8). Here we considered Src to be an input channel, and Tgt was an output channel.

For each X_*C*_ = X(θ, *C*), we estimated the probability function *p*(*C*) by histogramming the spike time arrays X_*C*_ with a fixed bin width of 0.01ms. The joint probability function *p*(*Src*, *Tgt*) of Src and Tgt was also estimated by histogramming all the spike time pairs of (X_*Src*_ X_*Tgt*_) with similar bin widths without any smoothing. We consider any missing spike (for example, cases when Src_i_ failed to induce a spikes in Tgt_i_ due to certain network configurations or parameter set) to take the value of max(X_*C*_) + 2σ(X_*C*_) to minimize distortions in the marginal distributions of both Src and Tgt. Removing those cases entirely led to misrepresentation of the marginal distribution and join distribution. For demonstration purposes, the value used for missing spikes was 1000 ms (Fig 8A_4_).

We calculated the mutual information between Src and Tgt with Eq 12 in which H(*A*) is the entropy of the distribution *p*(*A*) (Eq 13) and H(*A*, *B*) is the entropy of the joint distribution *p*(*A*, *B*) (Eq 14).

I(Src,Tgt)=H(Src)+H(Tgt)-H(Src,Tgt) (12)

$$H(A)=-\sum_{a}p(a){\text{log}}_{2}p(a)$$ (13)

$$H(A,\;B)=-\sum_{a,b}p(a,b){\text{log}}_{2}p(a,b)$$ (14)

We measured the transmission efficiency from the input channel (Src) to the output channel (Tgt) with Eq 15 [45]. This could be interpreted as % of the entropy of output that could be attributed to the input.

$$Transmission\;efficiency\;(Src\to Tgt)=\frac{I(Src,\;Tgt)}{H(Tgt)}\;\%$$ (15)

All code is available at https://github.com/jhaaslab/elec_ffwd_inh_circuit.
